# A Train-the-Trainer Point of Care Ultrasound (POCUS) Program for Pediatric Pneumonia in a Low-Resource Setting

**DOI:** 10.24908/pocusj.v10i01.18285

**Published:** 2025-04-15

**Authors:** Michelle S. Lee, Fatima Mir, Amerta Ladhani, Huba Atiq, Shaun K. Morris, Mark O. Tessaro

**Affiliations:** 1 Division of Emergency Medicine, Hospital for Sick Children, University of Toronto, ON, CAN; 2 Division of Infectious Diseases, Aga Khan University Hospital, Karachi, PAK; 3 Center for Excellence for Trauma & Emergencies, Aga Khan University Hospital, Karachi, PAK; 4 Division of Infectious Diseases, Hospital for Sick Children, University of Toronto, ON, CAN

**Keywords:** lung POCUS, resource-limited setting, POCUS training

## Abstract

**Background:**

Lung point of care ultrasound (POCUS) has the potential to transform pediatric pneumonia care in low resource settings. Prior studies of novice POCUS users in such settings showed high agreement with remote POCUS experts for diagnosing pediatric pneumonia, but use of remote experts may falsely inflate this agreement.

**Objectives:**

This study aimed to 1. Deliver a train-the-trainer program in Pakistan on lung POCUS for diagnosing pediatric pneumonia; 2. Determine inter-rater reliability between i) study-trained community health workers (CHWs) and a remote expert, with both interpreting POCUS examinations acquired by the CHWs, and ii) study-trained CHWs and local champions, with both interpreting examinations that they had acquired.

**Methods:**

Phase 1: Canadian pediatric POCUS experts developed and delivered a lung POCUS training program for two user groups in Pakistan. These groups included local champions (who had POCUS experience) and CHWs (who were POCUS novices). Phase 2: Children with suspected pneumonia underwent two lung POCUS examinations, one by a CHW and one by a local champion. Examinations were recorded and later reviewed by a remote expert for interpretation and quality assurance. Inter-rater reliability was determined.

**Results:**

Two local champions and three CHWs were successfully trained. An analysis of 231 recruited patients showed strong inter-rater reliability between study-trained CHWs and remote expert interpretations (κ = 0.83). In contrast, inter-rater reliability was moderate (κ = 0.66) between interpretations by novices and local champions when these users interpreted the examinations that they themselves had acquired.

**Conclusion:**

Our study showed that train-the-trainer programs are feasible and can be effective, while highlighting the importance of hands-on training and having local champions provide longitudinal support to novices.

## Background

Pediatric pneumonia remains a significant global health concern, especially in low-resource settings where accurate and timely diagnostic tools are limited [1–3]. Early and accurate pneumonia diagnosis is crucial for initiating appropriate treatment and reducing pediatric morbidity and mortality. In recent years, lung point of care ultrasound (POCUS) has emerged as a promising diagnostic modality for pediatric pneumonia, offering many advantages including being portable, lacking radiation and having a relatively low associated cost [4–7].

In low resource settings, general practitioner physicians are able to learn POCUS with brief focused training [8–11]. For pediatric lung POCUS, Pervaiz et al. [12] described a standardized program for general practitioners in Bangladesh that resulted in a sensitivity of 88% and a specificity of 92% for detecting lung consolidation when compared to remote expert review. Similarly, clinical officers performing pediatric lung POCUS for consolidation in South Sudan correctly interpreted 86% of examinations, as determined by remote expert review [13]. However, these remote expert review methods might artificially inflate estimates of agreement, as incomplete examinations by novice users might miss and not capture images of abnormalities. The promising results of these studies also contrast with the results of an inter-rater reliability study conducted in a tertiary care setting in North America, which reported only moderate agreement (κ = 0.56) for lung POCUS interpretations amongst POCUS-credentialed emergency physicians [14]. As POCUS use by novice primary care providers becomes widespread [15], further research is required to elucidate the efficacy and feasibility of the programs used to train them.

In Pakistan, pediatric acute respiratory tract infections are often diagnosed and managed by community health workers (CHWs) [16,17]. The CHW program began in 1994 as part of a national health improvement strategy in the face of significant economic, governmental, and security challenges [18]. CHWs use the World Health Organization (WHO) Integrated Management of Childhood Illnesses (IMCI) algorithm [19] in pediatric cases of acute respiratory infections, which uses simple clinical findings (respiratory rate, chest indrawing and danger signs) to diagnose pneumonia and to determine whether the patient can be treated at home with oral antibiotics or requires referral to a higher-level health care facility. The IMCI approach exhibits both poor sensitivity and specificity for pneumonia. This can result in both overuse of antibiotics in children without pneumonia and inadequate recognition of pneumonia cases requiring referral to higher-level care [20–22]. If POCUS can be effectively utilized at the primary care level, it could help users, such as CHWs, improve the detection and management of pediatric pneumonia.

## Objectives

The main objectives of our study were to:

1. deliver a multimodal train-the-trainer program in Pakistan for local champions (who have POCUS experience), who then trained CHWs (who had no POCUS experience) on POCUS use for diagnosing pediatric pneumonia.
2. determine inter-rater reliability between i) study-trained CHWs and a remote expert, both of whom interpreted POCUS examinations that had been acquired by the CHWs, and ii) study-trained CHWs and local champions, with both interpreting the examination that they had acquired.

Our unique study design involved both a novice user and a local champion consecutively acquiring POCUS examinations on the same patient. Since a more experienced POCUS user acquired and interpreted POCUS examinations, this approach may estimate agreement between users of different experience levels more accurately than studies employing remote expert review of novice-acquired examinations. The study also examined factors that may have contributed to disagreement between the novice POCUS users and local champions. By elucidating these pitfalls and predictable errors, future lung POCUS training programs can be strengthened.

## Methods

### Study design and settings

This was a feasibility study that took place at two hospitals in Karachi, Pakistan—Aga Khan University Hospital (AKUH) and Childlife Foundation Korangi Hospital. Researchers and pediatric lung POCUS experts at the Hospital for Sick Children in Toronto, Canada, collaborated on study design and materials. Ethical approval for the study was obtained from the research boards at the Hospital for Sick Children and AKUH.

During phase one of this study, two Canadian experts in pediatric lung POCUS (who had completed POCUS fellowship training) developed and refined a training program on lung POCUS for detecting pediatric pneumonia. This was delivered to study healthcare workers in Pakistan. In 2018, one of these experts delivered an in-person training program to two local POCUS champions at AKUH over the course of one week. This training standardized the local champions' knowledge on acquiring and interpreting lung POCUS examinations for diagnosing pediatric pneumonia and also included coaching for their role as lung POCUS trainers to study CHWs. The local champions translated the language of the teaching materials and adapted their content to cater to the CHWs. The following week, the local champions delivered the locally adapted training program to three CHWs at AKUH.

Phase two of the study was conducted from 2018-2019. Children presenting to participating hospitals with symptoms suggestive of pneumonia were recruited to undergo two consecutive lung POCUS examinations, one by a CHW and one by a local champion, such that inter-rater reliability could be determined.

### Phase 1 — Training program

The multi-modal training program comprised:

1. In-person teaching days—didactic session for two hours in the morning and hands-on practice for two hours in the afternoon. One day of teaching was delivered to local champions by the Canadian lung POCUS expert, and two days of teaching were delivered to CHWs by the local champions.
2. Mentored lung POCUS examinations—five examinations, with assessment and feedback using an Observed Structured Assessment of Technical Skills checklist (see Appendix 1) [23].
3. Computer-based exam—sixty lung POCUS video clips, where trainees interpreted the clips for normal or abnormal findings (passing score >80%).
4. Online lung ultrasound simulation and competency-based medical education platform (ImageSim)—with 100 cases showing a mixture of normal and abnormal ultrasound findings.
5. A total of 20 independently performed pediatric lung POCUS examinations—quality assurance (QA) was performed and feedback was given by remote experts.

#### Standardized training of local POCUS champions

As part of the study's train-the-trainer design, two local POCUS champions participated as trainers of the novice POCUS users. One was a pediatric emergency medicine physician with lung POCUS training and experience who routinely delivered POCUS training programs in Karachi. The other was a radiology technologist trained in lung POCUS in a previous research study. Both local champions had performed over 100 pediatric lung POCUS examinations prior to the study. For standardization purposes, both underwent the above training program over one week.

#### Adaptation of the training program by local POCUS champions

In collaboration with the on-site Canadian pediatric POCUS experts, the local champions adapted training materials for local CHW novice POCUS users. These adaptations included translating training materials from English to Urdu while incorporating local CHW clinical practice patterns and cultural considerations.

#### Standardized training of CHWs

Three CHWs who received routine medical training at AKUH volunteered to participate as novice POCUS users for this study. These CHWs had no prior POCUS training or experience. Immediately following the completion of the standardized training program of the local POCUS champions, the local champions delivered their adapted training program to the CHWs. Over the course of two weeks, the CHWs underwent all five components of the training program, delivered by the local champions, as listed above.

#### Lung POCUS examination protocol

The handheld ultrasound device used for this study consisted of a L12-4 linear transducer (Philips Lumify; Bothwell, WA) connected to an Android tablet running the Philips Lumify software application with the lung preset selected. Lung POCUS examinations employed a standard six zone protocol, with longitudinal and transverse views of each hemithorax at the mid-clavicular line, mid-axillary line, and posterior paraspinal line.24,25 Lung POCUS findings suggestive of pneumonia were defined as an area of subpleural consolidation with hepatization and a diameter greater than one centimeter, plus one or more of focal B lines, shredding, air or fluid bronchograms [24,25]. We acknowledged that other disease entities may have overlapping findings (such as atelectasis in asthmatics, Tuberculosis, etc.). Thus, during the training we always emphasized that the clinical context must be considered.

### Phase 2— Inter-rater reliability

This phase investigated the inter-rater reliability of lung POCUS for the detection of pediatric pneumonia between novice users and experienced users. The novice users (CHWs) were compared to both the local champions and to remote experts. The CHWs and the local champions each interpreted the examinations that they had acquired, recording their interpretations immediately after the conclusion of their POCUS examinations. At a later date, the remote experts interpreted the examinations acquired by both the CHWs and the local champions.

#### Patient selection

Patients presenting to AKUH or Childlife Foundation Korangi Hospital were eligible for recruitment if they were 2 months to <18-years-old and were presenting to the hospital with acute respiratory symptoms (cough, coryza, reported fast breathing, and/or fever). They also needed to meet at least one of the following criteria in the last 72 hours:

- physician diagnosis of pneumonia
- imaging (chest X-ray and/or computed tomography) findings of pneumonia
- classified as pneumonia by the WHO IMCI [19]
- abnormal respiratory physical exam findings by physician (respiratory distress, retractions, lower chest wall indrawing, auscultatory crackles, noisy breathing, and/or oxygen saturation <96%)

Patients were excluded if they had a chest tube or their guardians were unable to provide informed consent.

At both hospitals, patients were recruited from the ambulatory clinic, emergency department, pediatric ward, and intensive care unity. Eligible patients were enrolled in the study if their guardian(s) provided informed consent for study participation. Patient demographics and basic clinical information were recorded by research assistants.

#### POCUS examination—acquisition and interpretation

Participating patients underwent two consecutive lung POCUS examinations—one by a study-trained CHW and another by a local champion. For patient comfort, the duration of each of these examinations was limited to eight minutes. Each POCUS user was blinded to the results of the other POCUS examination and to the patient's diagnosis, as well as all other demographic and clinical information. Following the standard six zone protocol, a total of 12 video clips was recorded from each examination, with each clip labeled on screen with the corresponding hemithorax, zone, and transducer orientation. At the end of each examination, the POCUS user recorded their interpretation using a standardized checklist. The clinical management of these patients was not altered by study procedures as the treating team was blinded to the POCUS examinations.

POCUS examinations were uploaded to a secure server and, at a later date, were reviewed by a remote POCUS expert at the Hospital for Sick Children (Toronto, Canada). The remote expert was blinded to patient information and to the interpretations of the CHW and the local POCUS champion. The remote expert interpreted each examination and recorded their interpretation using the above standardized checklist. A second remote expert was available to deliberate on borderline cases.

After recording their interpretation, the remote POCUS expert was unblinded to the interpretations of the CHW or local champion. The remote expert then rated the quality of each POCUS examination using the American College of Emergency Physicians (ACEP) Quality Assurance Grading Scale for lung POCUS (a 5-point Likert scale). In cases where there was a discrepancy between the interpretations of the CHW and local champion, the remote expert recorded the reason for the discrepancy.

### Data and statistical analysis

Data analysis was conducted using IBM SPSS Statistics (Version 28; Armonk, NY). Patient demographic data were analyzed using descriptive statistics. Inter-rater reliability was reported using the weighted Cohen's kappa (κ) statistic.

## Results

Between November 2018 and March 2019, 333 patients were screened, 246 were enrolled, and 231 completed study procedures (the analysis population). The baseline characteristics of the analysis population are summarized in Table 1.

**Table 1.** Characteristics of the analysis population.

Demographic characteristics	Analysis Population (n = 231)
**Age group, n (%)**	
Age < 2y	166 (71.9)
Age ≥ 2y	65 (29.1)
**Sex (male), n (%)**	142 (61.4)
**Presenting symptoms, n (%)**	
Cough	228 (98.7)
Fever	186 (80.5)
Difficulty breathing	208 (90.0)
**Physical exam findings, n (%)**	
Increased work of breathing	163 (70.5)
Crackles	55 (23.8)
Wheeze	58 (25.1)
O2 saturation < 96%	47 (20.3)
Requiring O2	10 (4.3)
**Chest x-ray, n (%)**	
Available	125 (54.1)
Positive for pneumonia	89 (38.5)
Admission diagnosis*, n (%)	
Pneumonia	169 (73.2)
Bronchiolitis	51 (22.1)
Asthma	15 (6.5)
Other	17 (7.4)
**Discharge diagnosis, n (%)**	
Available	157 (68)
Pneumonia	81 (51.6)
Bronchiolitis	32 (20.4)
Asthma	21 (13.4)
Other	23 (14.6)

* Some patients had multiple diagnoses.

Table 2 displays the inter-rater reliability of POCUS interpretations by local POCUS users (CHW or local champion) and the remote expert. With the remote expert interpreting the locally acquired images, there appeared to be strong κ agreement of 0.83 (95% CI of 0.75 to 0.89) between remote expert and CHW, and κ of 0.90 (95% CI of 0.83 to 0.95) between remote expert and local champion.

**Table 2.** Inter-rater reliability of lung POCUS interpretation - local POCUS users vs remote expert

	Weighted Cohen's kappa (95% CI)
Remote expert vs combined CHWs	0.83 (0.75–0.89)
Remote expert vs CHW #1	0.77 (0.62–0.91)
Remote expert vs CHW #2	0.88 (0.70–0.99)
Remote expert vs CHW #3	0.81 (0.69–0.93)
Remote expert vs local champion	0.90 (0.83–0.95)

In contrast, Table 3 displays the inter-rater reliability of POCUS interpretations by CHWs and the local POCUS champion—who both acquired and interpreted their own set of images. In this case, there was moderate agreement between the community health workers and the local champion, with a κ of 0.66 (95% CI 0.55 – 0.75).

**Table 3.** Inter-rater reliability of lung POCUS interpretation - CHWs vs local POCUS champion

	Weighted Cohen's kappa (95% CI)
Local champion vs combined CHWs	0.66 (0.55–0.75)
Local champion vs CHW #1	0.63 (0.45–0.81)
Local champion vs CHW #2	0.76 (0.62–0.91)
Local champion vs CHW #3	0.57 (0.39–0.75)

Table 4 displays the remote expert quality ratings of the POCUS examinations obtained by the CHWs and local champions. ACEP Quality Assurance Grading Scale ratings of ≥3 are considered sufficient for diagnostic interpretation [26]. This was achieved in 214 (92.6%) of CHW examinations and 230 (99.5%) of local champion examinations.

**Table 4.** Remote expert scoring of lung POCUS examination quality using the ACEP Quality Assurance Grading Scale

ACEP Score	CHWs (all 3 aggregate)	Local champions
1	0	0
2	12	1
3	12	1
4	37	13
5	165	207
Mean	4.5	4.9

Table 5 displays discrepancy data regarding the interpretations of CHWs and local POCUS champions.

**Table 5.** POCUS interpretation discrepancies between CHWs and local POCUS champions

**Examinations with discrepant interpretations, n (% of total examinations)**	**36 (15.6)**
**Discrepancy type, n (% of total discrepancies)**	
CHW interpreted as *normal*, but pneumonia was captured by local champion	20 (55.6)
CHW interpreted as *pneumonia*, but local champion interpreted as normal	16 (44.4)
**Reason for discrepancy, n (% of total discrepancies)**	
Consolidation measurement error	10 (27.8)
Inadequate image acquisition technique	8 (22.2)
Normal anatomy interpreted as consolidation	6 (16.7)
Consolidation not captured	6 (16.7)
Consolidation interpreted as normal lung	4 (11.1)
Other	2 (5.5)

## Discussion

Our multi-modal train-the-trainer model of a pediatric lung POCUS training program for novice POCUS users was successfully implemented for a group of CHWs in Pakistan. With only brief training, our novice POCUS exhibited high rates of acquiring lung POCUS examinations of sufficient quality for diagnostic interpretation, while also displaying moderate agreement with local POCUS champions in lung POCUS interpretation. These results suggest that training programs such as ours are feasible and effective.

Notably, our inter-rater reliability results differed depending on whether the novice POCUS interpretations were compared to a local champion who also scanned the patient versus a remote expert reviewing the novice-acquired images. Inter-rater reliability was high when interpretations were compared between local POCUS users and the remote expert, which is consistent with prior studies that relied on remote POCUS expert review [12,13]. In contrast, we found only moderate inter-rater reliability when comparing the interpretations of CHWs with those of local POCUS champions. One plausible explanation for this difference is that consolidations that were not captured in POCUS examinations by novice users would also not be detected during remote expert interpretation of these examinations. If the local POCUS champion performed a more thorough examination that allowed them to capture such consolidations, there would be lower rates of agreement between the novice users and local champion. In other words, our results indicated that inter-rater reliability for pediatric lung POCUS interpretation may be falsely inflated if we rely solely on remote expert review and support the importance of local POCUS champions in these types of studies.

Remote expert review in this study did, however, allow us to explore reasons for discrepancies between CHWs and local POCUS champions. Our results suggest that high yield areas of focus for improving similar future training programs are techniques for image acquisition and consolidation measurement (focusing on caliper placements), as well as distinguishing normal anatomy (such as stomach and diaphragm) from consolidation.

Our study has several limitations. Our analysis would be more robust with more novice POCUS users. Unfortunately, this was not possible at the time of the study due to resource constraints in the local setting. We also did not perform an analysis of the diagnostic accuracy of the different POCUS user groups, as we were unable to apply a consistent diagnostic gold standard for pneumonia across our analysis population. There is already, however, a robust body of literature demonstrating that lung POCUS is as accurate as chest X-ray for detecting pediatric pneumonia in diverse settings [4,27]. We also only had the linear probe available, which may have been challenging to use in teenagers with larger body habitus. Finally, although our multi-modal training program was feasible and effective in this particular setting, a program with multiple training days for novice POCUS users may not be generalizable to other low-resource settings.

## Conclusion

Lung POCUS for the detection of pediatric pneumonia has previously proven to be useful in diverse settings and adoption of this technology to new user groups is steadily increasing. Our study suggests that it can feasibly and effectively be adopted by CHWs with no prior POCUS experience after a brief multi-modal training program. Our results also highlight the importance of hands-on training and suggest high-yield areas of focus to improve similar training programs in the future. Finally, our findings indicate the importance of having local POCUS champions provide longitudinal support to novice users, rather than relying solely on remote POCUS experts. Additional large-scale research is required to further examine the best approach for designing, implementing, and sustaining train-the-trainer POCUS programs for novice users in low-resource settings.
